# Efficacy and Safety of Botulinum Toxin Type A for Limb Spasticity after Stroke: A Meta-Analysis of Randomized Controlled Trials

**DOI:** 10.1155/2019/8329306

**Published:** 2019-04-07

**Authors:** Li-Chun Sun, Rong Chen, Chuan Fu, Ying Chen, Qianli Wu, RuiPeng Chen, XueJuan Lin, Sha Luo

**Affiliations:** ^1^Department of Rehabilitation Medicine, The First Affiliated Hospital of Hainan Medical University, Haikou, Hainan, 570102, China; ^2^Department of Neurology, The First Affiliated Hospital of Hainan Medical University, Haikou, Hainan, 570102, China; ^3^Department of Medical Ultrasonics, The First Affiliated Hospital of Hainan Medical University, Haikou, Hainan, 570102, China

## Abstract

**Background:**

Inconsistent data have been reported for the effectiveness of intramuscular botulinum toxin type A (BTXA) in patients with limb spasticity after stroke. This meta-analysis of available randomized controlled trials (RCTs) aimed to determine the efficacy and safety of BTXA in adult patients with upper and lower limb spasticity after stroke.

**Methods:**

An electronic search was performed to select eligible RCTs in PubMed, Embase, and the Cochrane library through December 2018. Summary standard mean differences (SMDs) and relative risk (RR) values with corresponding 95% confidence intervals (CIs) were employed to assess effectiveness and safety outcomes, respectively.

**Results:**

Twenty-seven RCTs involving a total of 2,793 patients met the inclusion criteria, including 16 and 9 trials assessing upper and lower limb spasticity cases, respectively. For upper limb spasticity, BTXA therapy significantly improved the levels of muscle tone (SMD=-0.76; 95% CI -0.97 to -0.55; P<0.001), physician global assessment (SMD=0.51; 95% CI 0.35-0.67; P<0.001), and disability assessment scale (SMD=-0.30; 95% CI -0.40 to -0.20; P<0.001), with no significant effects on active upper limb function (SMD=0.49; 95% CI -0.08 to 1.07; P=0.093) and adverse events (RR=1.18; 95% CI 0.72-1.93; P=0.509). For lower limb spasticity, BTXA therapy was associated with higher Fugl-Meyer score (SMD=5.09; 95%CI 2.16-8.01; P=0.001), but had no significant effects on muscle tone (SMD=-0.12; 95% CI -0.83 to 0.59; P=0.736), gait speed (SMD=0.06; 95% CI -0.02 to 0.15; P=0.116), and adverse events (RR=1.01; 95% CI 0.71-1.45; P=0.949).

**Conclusions:**

BTXA improves muscle tone, physician global assessment, and disability assessment scale in upper limb spasticity and increases the Fugl-Meyer score in lower limb spasticity.

## 1. Introduction

Stroke is characterized by sudden development of signs of focal and global cerebral function disturbance lasting more than 24 hours or leading to death. Currently, stroke has high morbidity, mortality, and disability and constitutes one of the top three causes of death in China [1, 2]. Further, stroke causes various degrees of disability in approximately 90% of patients, and nearly 1/4 individuals that experience a stroke develop recurrent stroke within a few weeks or months [3, 4]. Spasticity is an important cause of dysfunction and is observed in 19% of patients within 3 months and 38% within 12 months [5, 6]. Spasticity could affect rehabilitation and cause muscle and joint atrophy, which results in shortening of muscle fibers and ligaments, and hinder improvement in daily living activities [7]. However, few effective treatments are available for stroke and sequelae.

Botulinum toxin type A (BTXA) is a potent neurotoxin from the bacterium* Clostridium botulinum*, which blocks acetylcholine release at neuromuscular junctions. Numerous studies reported local injection of BTXA improves muscle tone, motion, and pain [8–13]. However, the therapeutic effects are often transient and vary by BTXA dose, for which no guidelines offer a unified standard [14]. However, injections of BTXA are widely used in patients with obvious spasticity and after 3 months in stroke patients, which might affect their rehabilitation process [15, 16]. Further, the treatment effectiveness of BTXA in upper or lower limb spasticity according to different follow-up durations and other patient characteristics remains limited and inconclusive.

Recently, numerous randomized controlled trials (RCTs) have investigated the efficacy and safety of BTXA for upper or lower limb spasticity after stroke and reported inconsistent findings. Clarifying the optimal treatment effects is particularly important in patients with limb spasticity. Therefore, we performed a large-scale analysis of available RCTs to determine the efficacy and safety of BTXA in patients with upper or lower limb spasticity. Furthermore, whether treatment effectiveness differs according to study or patient characteristics was assessed using metaregression and subgroup analyses.

## 2. Material and Methods

### 2.1. Data Sources, Search Strategy, and Selection Criteria

This review was performed and reported according to the Preferred Reporting Items for Systematic Reviews and Meta-Analysis Statement issued in 2009 [17]. RCTs that investigated the efficacy and safety of BTXA for upper or lower limb spasticity after stroke were included in this study. The core search terms “botulinum toxin, type A” and “spasticity” were used to query the PubMed, Embase, and Cochrane library databases through December 2018. The detailed search strategies in PubMed are shown in Supplementary 1. A manual search of reference lists of relevant reviews and studies was also carried out to identify additional potential studies for inclusion.

The study selection process was independently undertaken by 2 investigators (Li-Chun Sun and Ying Chen), and any disagreement was resolved by group discussion until a consensus was reached. A study was included if the following inclusion criteria were met: (1) patients with upper or lower spasticity after stroke included; (2) BTXA as intervention; (3) control group administered a placebo; (4) outcomes as muscle tone, active upper limb function, physician global assessments, disability assessment scale, and adverse events for upper spasticity, and muscle tone, Fugl-Meyer score, gait speed, and adverse events for lower limb spasticity; (5) study design as RCT.

### 2.2. Data Collection and Quality Assessment

Data collection and quality assessment were performed by 2 authors (Rong Chen and Chuan Fu), and inconsistencies were examined and adjudicated by group discussion referring to the original studies. The information collected included first author's surname, publication year, country, sample size, mean patient age, percentage of males, time since event, spasticity sites, dose of BTXA, injection technique, duration of follow-up, and reported outcomes. Study quality was evaluated by the JADAD scale, based on randomization, concealment of treatment allocation, blinding, completeness of follow-up, and the use of intention-to-treat analysis [18]. The scoring system ranged from 0 (low quality) to 5 (high quality) in study quality assessment.

### 2.3. Statistical Analysis

Efficacy results of individual trials were assigned as continuous data, and safety results as dichotomous frequency data. Individual standard mean differences (SMDs) and relative risk (RR) values with 95% confidence intervals (CIs) were calculated from means, standard deviations, sample sizes, or event numbers. Summary effect estimates were calculated using the random-effects model since the actual underlying effect varies across included trials [19, 20]. Heterogeneity among the included trials was assessed by the I-square and Q tests; P values of the Q test < 0.10 were checked for statistical significance [21, 22]. Sensitivity analysis of the investigated outcomes was performed to evaluate the impact of each single trial on the overall findings [23]. The source of heterogeneity in estimates of efficacy results was identified by univariate metaregression [24]. Subgroup analyses of efficacy results were performed according to publication year, mean age, percentage male, time since event, follow-up duration, and study quality. Publication bias for the investigated outcomes was assessed by the Egger [25] and Begg tests [26]. P values in pooled analysis are two-sided, and a value below 0.05 was considered statistically significant. All statistical analyses were performed with the Stata software (version 10.0; Stata Corporation, College Station, TX, USA).

## 3. Results

### 3.1. Literature Search

**Figure 1:**
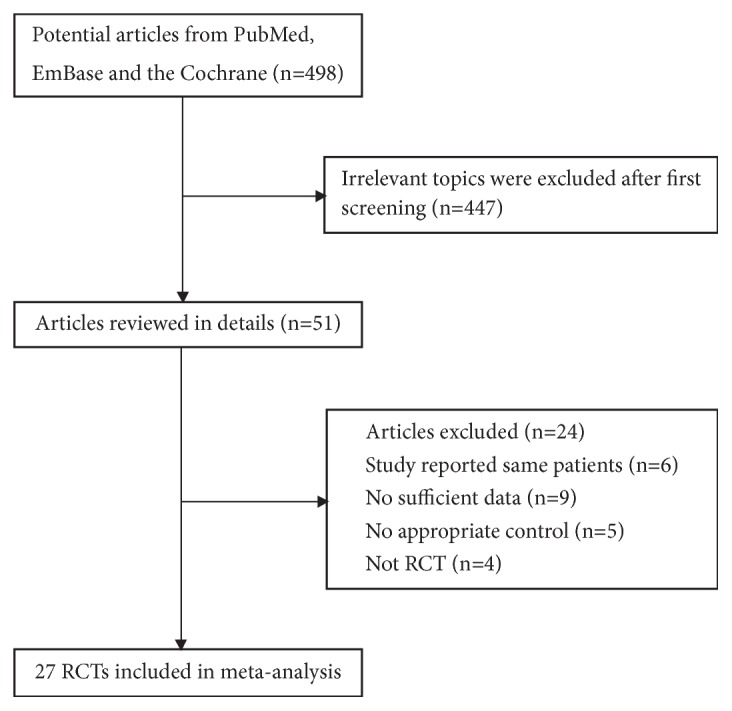
The literature search and study selection process.

The electronic search of electronic databases produced 498 articles, and 447 studies were excluded for irrelevance. The remaining 51 were selected for further evaluation, and 27 RCTs involving 2,793 spasticity patients were selected in the final analysis [27–53]. The manual search of reference lists from relevant reviews and these 27 studies yielded no additional eligible studies. The literature search and study selection are presented in Figure 1, and the baseline characteristics of included studies are shown in Table 1.

### 3.2. Study Characteristics

Of the 27 included trials, 18 were conducted in patients with upper limb spasticity [27–44], and the remaining 9 studies in individuals with lower limb spasticity [45–53]. Studies were published from 1996 to 2018, with 20-468 patients included in each trial. Twenty of the included trials were conducted in Western countries, 6 in Eastern countries, and the remaining 1 in multiple countries [53]. The mean patient age ranged from 49.3 to 63.5 years, with a percentage of males ranging from 40.0 to 80.0. Study quality was evaluated using the JADAD scale; 10 trials had a score of 5, 8 had 4 points, 8 had 3 points, and the remaining 1 had 2 points.

### 3.3. Upper Limb Spasticity

**Figure 2:**
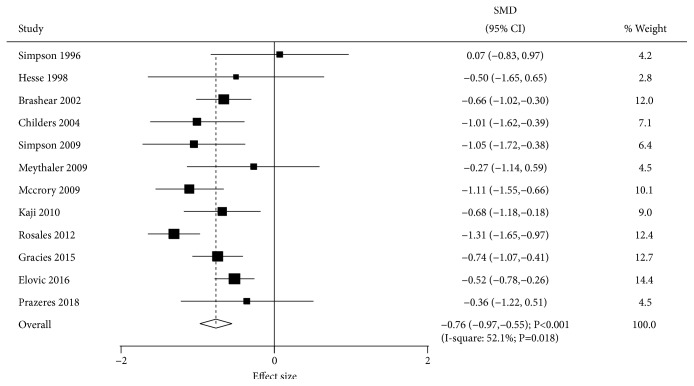
Effect of BTXA on muscle tone in upper limb spasticity.

Data for the effect of BTXA on muscle tone were available in 12 trials. The summary SMD indicated that BTXA therapy was associated with lower muscle tone compared with the placebo group (SMD=-0.76; 95% CI -0.97 to -0.55; P<0.001; Figure 2). There was significant heterogeneity among these trials (I-square, 52.1%; P=0.018). Sensitivity analysis indicated the results were not altered by sequentially excluding individual trials. Univariable metaregression analyses indicated percentage of males (P=0.011) and time since event (P=0.006) altered BTXA's effects on muscle tone. Subgroup analysis indicated significant differences between BTXA and placebo for muscle tone in most subsets, while no significant differences between BTXA and placebo groups were observed for muscle tone for a duration of follow-up > 12 weeks and in low quality studies (Table 2).

**Figure 3:**
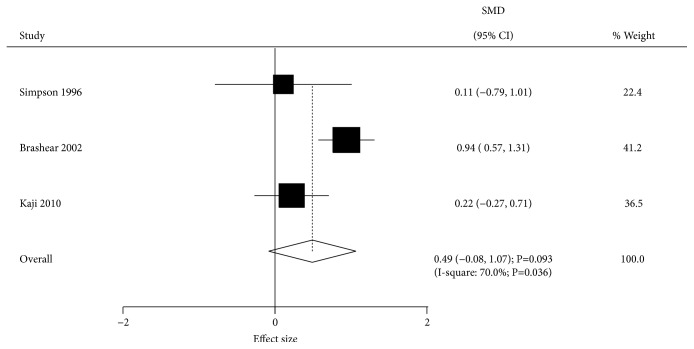
Effect of BTXA on active upper limb function in upper limb spasticity.

The effect of BTXA on active upper limb function was assessed by 3 trials. There was no significant difference between BTXA and placebo groups for active upper limb function (SMD=0.49; 95% CI -0.08 to 1.07; P=0.093; Figure 3). Significant heterogeneity was recorded across the included trials (I-square, 70.0%; P=0.036). Sensitivity analysis indicated that the lack of significant difference was not changed after excluding any particular trial. Univariable metaregression analysis indicated publication year (P=0.049), percentage male (P=0.049), and follow-up duration (P=0.015) could bias the effect of BTXA on active upper limb function. Subgroup analysis indicated BTXA therapy was associated with high active upper limb function with a follow-up of 4 or 6 weeks (Table 2).

**Figure 4:**
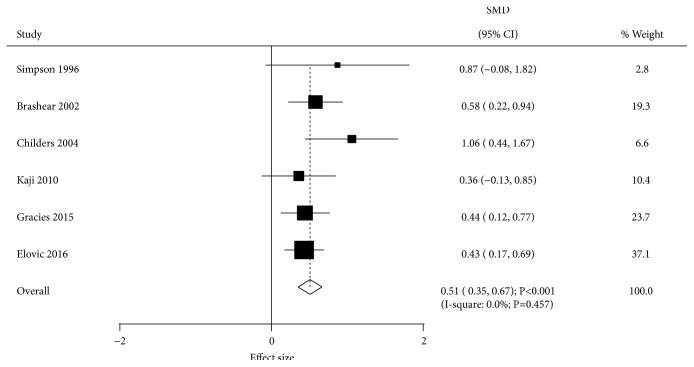
Effect of BTXA on physician global assessments in upper limb spasticity.

Data reporting the effect of BTXA on physician global assessment were available in 6 trials. BTXA therapy significantly increased physician global assessment compared with the placebo group (SMD-0.51; 95% CI 0.35 to 0.67; P<0.001; Figure 4). There was no evidence of heterogeneity among the included trials (I-square, 0.0%; P=0.457). Metaregression analysis indicated follow-up duration could affect the treatment effect of BTXA (P=0.002). Subgroup analysis indicated no significant difference between BTXA and placebo for physician global assessment when pooling low quality studies (Table 2).

**Figure 5:**
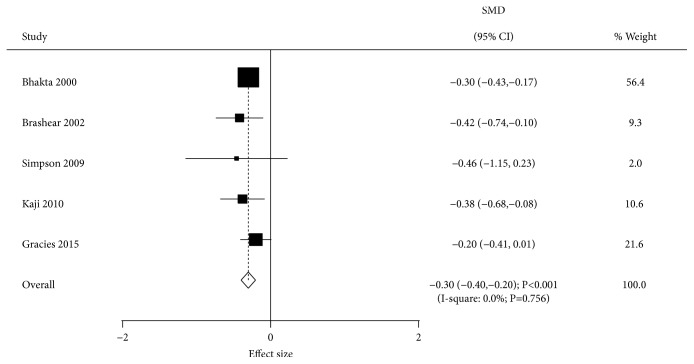
Effect of BTXA on disability assessment scale in upper limb spasticity.

The effect of BTXA on disability assessment scale was evaluated in 5 trials. BTXA therapy significantly reduced this scale compared with placebo administration (SMD=-0.30; 95% CI -0.40 to -0.20; P<0.001; Figure 5). No evidence of heterogeneity across the included trials was detected (I-square, 0.0%; P=0.756). Univariable metaregression analysis indicated no factors affecting the treatment effect of BTXA. Subgroup analysis indicated significant effects of BTXA in most subsets, with no significant difference in disability assessment scale for time since event < 24.0 months (Table 2).

**Figure 6:**
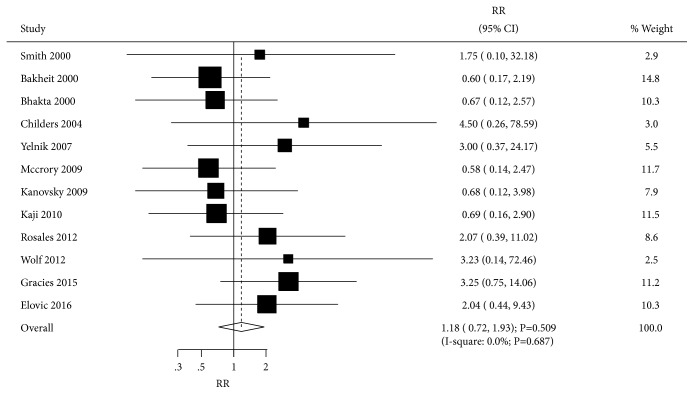
Effect of BTXA on adverse events in upper limb spasticity.

Data reporting the effect of BTXA on adverse events were available in 12 trials. BTXA therapy had no significant effect on the risk of adverse events compared with the placebo group (RR=1.18; 95% CI 0.72 to 1.93; P=0.509; Figure 6). There was no evidence of heterogeneity among the included trials (I-square, 0.0%; P=0.687), and the summary result was robust.

### 3.4. Lower Limb Spasticity

**Figure 7:**
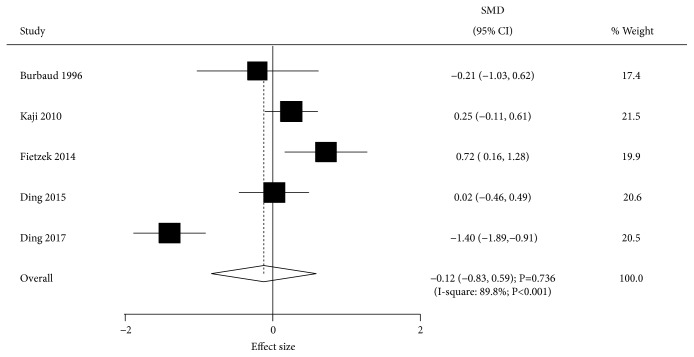
Effect of BTXA on muscle tone in lower limb spasticity.

Data describing the effect of BTXA on muscle tone were available in 5 trials. BTXA had no significant effect on muscle tone compared with the placebo group (SMD=-0.12; 95% CI -0.83 to 0.59; P=0.736; Figure 7). Substantial heterogeneity across the included trials was observed (I-square, 89.8%; P<0.001). In sensitivity analysis, the conclusion was not altered after trials were sequentially excluded from the pooled results. Mean age (P<0.001), percentage of males (P=0.037), follow-up duration (P=0.049), and study quality (P<0.001) affected the effect of BTXA on muscle tone as assessed by univariable metaregression analysis. Subgroup analysis indicated BTXA could increase muscle tone after 4 weeks of follow-up (Table 3).

**Figure 8:**
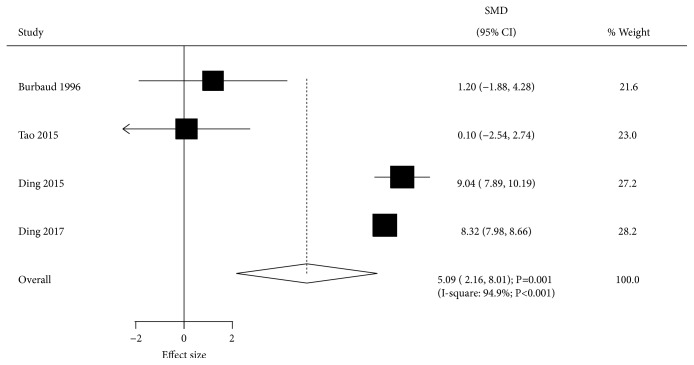
Effect of BTXA on Fugl-Meyer score in lower limb spasticity.

Data describing the effect of BTXA on Fugl-Meyer score were available in 4 trials. The summary result indicated that BTXA significantly increased Fugl-Meyer score compared with the placebo group (SMD=5.09; 95% CI 2.16 to 8.01; P=0.001; Figure 8). Substantial heterogeneity among the included trials was observed (I-square, 94.9%; P<0.001), and the results were stable after excluding individual trials. Univariable metaregression analysis suggested that publication year (P<0.001), mean age (P<0.001), percentage of males (P<0.001), time since event (P<0.001), and follow-up duration (P<0.001) could bias the effect of BTXA on Fugl-Meyer score. Subgroup analysis indicated no significant differences between BTXA and placebo in Fugl-Meyer score when pooling trials published before 2010, as well as those with mean patient age <55.0 years, percentage of males ≥60.0%, time since event ≥24.0 months, and more than 4 weeks of follow-up (Table 3).

**Figure 9:**
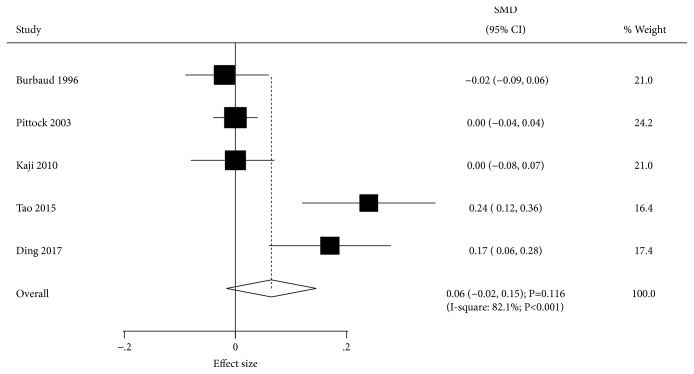
Effect of BTXA on gait speed in lower limb spasticity.

Data describing the effect of BTXA on gait speed were available in 5 trials. There was no significant difference between BTXA and placebo for gait speed (SMD=0.06; 95% CI -0.02 to 0.15; P=0.116; Figure 9). Although significant heterogeneity among the included trials was detected (I-square, 82.1%; P<0.001), the conclusion was not altered by the exclusion of any specific study. Univariable metaregression analysis indicated that publication year (P=0.003), percentage of males (P=0.016), and study quality (P=0.014) could bias the effect of BTXA on gait speed. BTXA therapy significantly increased gait speed with percentage of males < 60.0% (Table 3).

**Figure 10:**
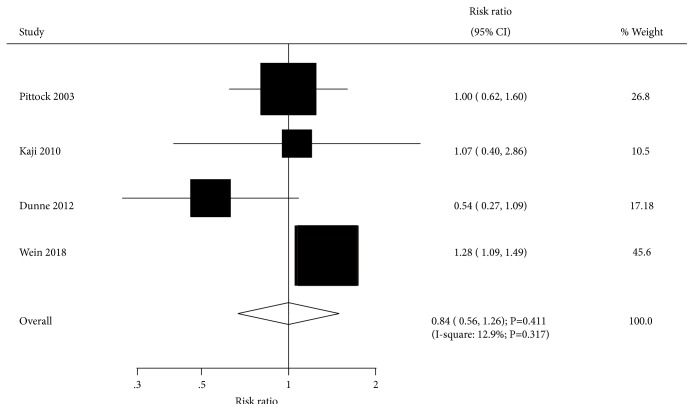
Effect of BTXA on adverse events in lower limb spasticity.

The effect of BTXA on adverse events was assessed by 4 trials. Overall, BTXA therapy had no significant effect on the risk of adverse events (RR=1.01; 95% CI 0.71-1.45; P=0.949; Figure 10). There was potential significant heterogeneity across the included trials (I-square, 53.6%; P=0.091), and sensitivity analysis indicated the conclusion was not affected by sequential exclusion of individual trials.

### 3.5. Publication Bias

Publication bias was assessed for the investigated outcomes, and results are shown in Table 4. There were no significant publication biases for muscle tone (Egger's P value=0.591; Begg's P value=0.409), active upper limb function (Egger's P value=0.467; Begg's P value=1.000), physician global assessment (Egger's P value=0.136; Begg's P value=0.133), disability assessment scale (Egger's P value=0.370; Begg's P value=0.462), and adverse events (Egger's P value=0.081; Begg's P value=0.064) in patients with upper limb spasticity. Furthermore, no evidence of publication bias was found for muscle tone (Egger's P value=0.838; Begg's P value=0.806), Fugl-Meyer score (Egger's P value=0.226; Begg's P value=0.308), and adverse events (Egger's P value=0.209; Begg's P value=0.734) in patients with lower limb spasticity. Although the Egger's test suggested no publication bias for gait speed (P=0.136), potential publication bias was detected in the Begg's test in patients with lower limb spasticity (P=0.043). The conclusion regarding gait speed was not altered after adjustment by the trim and fill method [54].

## 4. Discussion

BTXA is widely used for limb spasticity, but its effectiveness varies according to spasticity site and treatment dose. The aim of this study was to determine the efficacy and safety of BTXA therapy for upper and lower limb spasticity after stroke. In this comprehensive quantitative meta-analysis, 27 RCTs involving 2,793 spasticity patients with a broad range of characteristics could ensure the applicability of summary findings. Pooled results showed that BTXA therapy could significantly improve muscle tone, physician global assessment, and disability assessment scale in upper limb spasticity compared with the placebo group. In addition, BTXA versus placebo showed significant improvement in Fugl-Meyer score in lower limb spasticity. Finally, the treatment effects of BTXA might be affected by several predefined factors, including publication year, mean age, percentage of males, time since event, follow-up duration, and study quality.

A previous meta-analysis has investigated the efficacy and safety of BTXA for upper limb spasticity after stroke and traumatic brain injury [55]. The authors concluded BTXA therapy has beneficial effects with improved muscle tone, reduced disability assessment scale, and increased patients' global assessment score. In addition, they pointed out that BTXA therapy is well-tolerated in patients with upper limb spasticity after stroke. Another meta-analysis was performed in patients with lower limb spasticity and concluded BTXA therapy produces persistent benefits for muscle tone and Fugl-Meyer score compared with placebo administration [56]. The limitations of previous meta-analyses are that they just provide summary effect estimates according to the follow-up duration and in-depth stratified analyses are not illustrated. Therefore, the current meta-analysis was performed to determine the efficacy and safety of BTXA in patients with upper and lower limb spasticity and evaluate whether the treatment effectiveness of BTXA differs according to publication year, mean age, percentage male, time since event, follow-up duration, and study quality in upper and lower limb spasticity, respectively.

Summary results indicated BTXA was superior to placebo for upper limb spasticity in terms of muscle tone, physician global assessment, and disability assessment scale. Indeed, most included trials reported significant differences between BTXA and placebo in muscle tone, physician global assessment, and disability assessment scale, while several others found no statistically significant differences [27, 28, 36, 44]. A possible explanation might be the small sample sizes of these trials, which showed large standard deviations and broad 95% CIs. In addition, muscle tone assessment is highly subjective, with varying indexes among the included trials, which might produce potential bias. Furthermore, although summary results for active upper limb function were not statistically significant, an obvious trend of increase was detected, which requires further large-scale trials for verification. Finally, the risk of adverse events between BTXA and placebo showed no statistically significant difference, and all the included trials reported similar conclusions. This could be explained by an incidence of adverse events lower than expected as well as broad 95% CI.

As shown above, BTXA could improve Fugl-Meyer score, but had no significant effect on muscle tone, gait speed, and adverse events in patients with lower limb spasticity after stroke. First, only 2 of the included trials reported significant effects of BTXA on muscle tone, with discrepant results. Fietzek et al. indicated patients administered BTXA show high muscle tone compared with the placebo group [49]. This might be because muscle tone deterioration was observed in patients who initially had placebo, which is associated with reduced susceptibility to BTXA therapy. Secondly, pooled results for Fugl-Meyer score showed a significant increase in patients administered BTXA since the study by Ding et al. reported a large effect difference between the BTXA and placebo groups [51, 52]. This might be explained by large sample sizes in these two studies and sufficient power to detect statistical significance. Furthermore, these two patients received electrical nerve stimulation, which could affect the Fugl-Meyer score. Thirdly, gait speed results between BTXA and placebo had no statistically significant difference, while two trials performed in China reported inconsistent findings. Further large-scale trials are required for verification. Finally, pooled results for adverse events showed no statistically significant difference, likely because of low incidence of events and small sample sizes of the included trials.

Subgroup analysis for upper or lower limb spasticity could be affected by predefined factors. Firstly, publication year affected active upper limb function in upper limb spasticity, as well as Fugl-Meyer score and gait speed in lower limb spasticity. This might be because studies performed at different periods could affect the level of background rehabilitation therapy. Secondly, mean patient age significantly biased results for muscle tone and Fugl-Meyer score in lower limb spasticity. The recovery ability in different age groups varies, which might affect the effects of BTXA. Thirdly, the percentage of males mostly affected efficacy results, likely because mean age varied between men and women among the included trials. Fourthly, time since event in patients administered BTXA could bias results for muscle tone in upper limb spasticity and Fugl-Meyer score in lower limb spasticity. This indicates that time of BTXA administration could affect treatment effects. Fifthly, the duration of follow-up reflected the persistent effects of BTXA and affected most results. Treatment effects were tapered after prolonged follow-up, especially after 12 weeks. Finally, study quality affected the balance of patients' characteristics, which plays an important role in the treatment effects of BTXA.

The limitations of this study should be mentioned. Firstly, the effects of BTXA on limb spasticity based on dose were not assessed due diverse units and types. Secondly, effect estimates had large ranges and/or different units; therefore, weighted mean differences could not be calculated. Thirdly, stratified analyses based on several important factors were not conducted due to smaller number of included trials for each specific outcome, such as country, injection techniques, and rating scales. Fourthly, this study was not registered online, which might be a potential shortcoming. Finally, inherent limitations of meta-analyses include publication bias and use of pooled results.

## 5. Conclusion

In conclusion, pooled results indicated BTXA is superior to placebo in upper or lower limb spasticity after stroke. However, the treatment effects taper after 12 weeks. Furthermore, publication year, mean patient age, percentage of males, time since event, and study quality could significantly affect efficacy results in patients administered BTXA. Future large-scale RCTs should be performed to verify the current findings and assess treatment effects according to patients' characteristics.

## Figures and Tables

**Table 1:** Baseline characteristics of studies included in the current systematic review and meta-analysis.

Study	Country	Sample size	Mean age	Percentage male (%)	Time since event	Spasticity sites	Dose of BTXA	Injection technique	Duration of follow-up	Jadad score
Simpson 1996 [27]	US	37	59.0	43.2	37.0 months	Upper	Botox 75, 150, 300 units	EMG	16.0 weeks	2

Hesse 1998 [28]	Germany	24	52.3	79.2	7.5 months	Upper	Dysport 1000 units	EMG	12.0 weeks	3

Smith 2000 [29]	UK	25	51.9	40.0	>12.0 months	Upper	Dysport 500, 1000, 1500 units	NA	12.0 weeks	4

Bakheit 2000 [30]	UK	83	62.5	62.7	>3.0 months	Upper	Dysport 500, 1000, 1500 units	Anatomical landmarks	16.0 weeks	4

Bhakta 2000 [31]	UK	40	57.0	57.5	37.2 months	Upper	Dysport 1000 units	Anatomical landmarks	12.0 weeks	5

Brashear 2002 [32]	US	126	61.5	50.0	56.4 months	Upper	Botox 200-240 units	NA	12.0 weeks	4

Childers 2004 [33]	US	91	60.0	67.0	25.8 months	Upper	Botox 90, 180, 360 units	EMG	9.0 weeks	5

Yelnik 2007 [34]	France	20	54.1	75.0	17.0 months	Upper	Dysport 500 units	ES	4.0 weeks	3

Simpson 2009 [35]	US	39	51.9	53.8	> 24 hours	Upper	Botox 500 units	ES	6.0 weeks	4

Meythaler 2009 [36]	US	21	53.3	71.4	> 6.0 months	Upper	Botox 300-400 units	EMG	12.0 weeks	4

Mccrory 2009 [37]	Australia	96	59.6	60.4	70.8 months	Upper	Dysport 750-1000 units	EMG or ES	20.0 weeks	5

Kanovsky 2009 [38]	Germany	148	55.6	64.2	55.0 months	Upper	Botox 400 units	EMG	12.0 weeks	5

Kaji 2010 [39]	Japan	109	63.2	67.9	82.9 months	Upper	Botox 120-150, 200-240 units	EMG nerve stimulator	12.0 weeks	5

Rosales 2012 [40]	Hong Kong, Malaysia, the Philippines, Singapore, and Thailand	163	55.1	66.9	1.7 months	Upper	Botox 500 units	NA	24.0 weeks	5

Wolf 2012 [41]	US	25	49.3	60.0	3.0-24.0 months	Upper	Dysport 300 units	NA	12.0 weeks	3

Gracies 2015 [42]	Europe and the US	238	52.8	64.3	61.2 months	Upper	Dysport 500, 1000 units	ES	12.0 weeks	5

Elovic 2016 [43]	US	317	56.0	46.4	28.0 months	Upper	Xeomin 400 units	EMG and/or ES ultrasound	4.0 weeks	5

Prazeres 2018 [44]	Brazil	40	52.3	60.0	33.1 months	Upper	Dysport NA	NA	24.0 weeks	4

Burbaud 1996 [45]	France	23	52.5	69.6	23.5 months	Lower	Dysport 1000 units	EMG	12.0 weeks	3

Pittock 2003 [46]	Ireland	234	55.5	NA	> 3.0 months	Lower	Dysport 500, 1000, 1500 units	NA	12.0 weeks	4

Kaji 2010 [47]	Japan	120	62.5	80.0	> 6.0 months	Lower	Botox 300 units	EMG	12.0 weeks	4

Dunne 2012 [48]	Australia	83	58.4	76.5	40.8 months	Lower	Botox 200, 300 units	EMG or ES	12.0 weeks	5

Fietzek 2014 [49]	Germany	52	54.3	57.7	< 3.0 months	Lower	Botox 230 units	NA	12.0 weeks	4

Tao 2015 [50]	China	23	56.5	65.2	< 1.5 months	Lower	Botox 200 units	EMG	8.0 weeks	3

Ding 2015 [51]	China	68	63.5	48.5	15.9 months	Lower	Lanzhou Institute of Biological Products 100 units/ampule	Electrical nerve stimulator	24.0 weeks	3

Ding 2017 [52]	China	80	61.9	51.3	4.2 months	Lower	Lanzhou Institute of Biological Products 350 units	Electrical nerve stimulator	12.0 weeks	3

Wein [53]	North America, Europe, Russia, the United Kingdom, 66 and Asia 5	468	56.5	64.7	64.3 months	Lower	Botox ≤ 400 units	EMG and/or ES ultrasound	6.0 weeks	5

*∗*EMG: electromyography; ES: electrical stimulation; NA: not available; UK: United Kingdom; US: United States.

**Table 2:** Subgroup analysis for upper limb spasticity.

Outcomes	Factors	Groups	SMD and 95% CI	P value	Heterogeneity (%)	P value for heterogeneity	P value for meta-regression
Muscle tone	Publication year	Before 2010	-0.76 (-1.04 to -0.48)	<0.001	31.0	0.192	0.909
		2010 or after	-0.77 (-1.10 to -0.43)	<0.001	71.9	0.007	
	Mean age (year)	≥ 55.0	-0.80 (-1.10 to -0.50)	<0.001	69.7	0.003	0.528
		< 55.0	-0.70 (-0.96 to -0.44)	<0.001	0.0	0.598	
	Percentage male (%)	≥60.0	-0.87 (-1.12 to -0.62)	<0.001	42.4	0.096	0.011
		<60.0	-0.59 (-0.86 to -0.32)	<0.001	30.0	0.232	
	Time since event (months)	≥24.0	-0.69 (-0.87 to -0.50)	<0.001	26.1	0.220	0.006
		<24.0	-0.93 (-1.42 to -0.44)	<0.001	50.5	0.109	
	Follow-up duration (weeks)	4	-0.98 (-1.28 to -0.68)	<0.001	65.8	0.005	0.231
		6	-0.83 (-1.17 to -0.49)	<0.001	20.0	0.287	
		8	-0.87 (-1.15 to -0.59)	<0.001	0.0	0.710	
		12	-0.62 (-0.83 to -0.42)	<0.001	0.0	0.538	
		> 12	-0.55 (-1.30 to 0.20)	0.152	69.0	0.040	
	Study quality	High	-0.81 (-1.02 to -0.60)	<0.001	53.4	0.023	0.079
		Low	-0.15 (-0.86 to 0.56)	0.685	0.0	0.444	

Active upper limb function	Publication year	Before 2010	0.63 (-0.16 to 1.42)	0.116	64.2	0.095	0.049
		2010 or after	0.22 (-0.27 to 0.71)	0.379	-	-	
	Mean age (year)	≥ 55.0	0.49 (-0.08 to 1.07)	0.093	70.0	0.036	-
		< 55.0	-	-	-	-	
	Percentage male (%)	≥60.0	0.22 (-0.27 to 0.71)	0.379	-	-	0.049
		<60.0	0.63 (-0.16 to 1.42)	0.116	64.2	0.095	
	Time since event (months)	≥24.0	0.49 (-0.08 to 1.07)	0.093	70.0	0.036	-
		<24.0	-	-	-	-	
	Follow-up duration (weeks)	4	0.55 (0.28 to 0.83)	<0.001	0.0	0.685	0.015
		6	1.11 (0.46 to 1.77)	0.001	72.5	0.026	
		12	0.19 (-0.24 to 0.63)	0.375	0.0	0.833	
	Study quality	High	0.60 (-0.11 to 1.30)	0.096	81.1	0.022	0.239
		Low	0.11 (-0.79 to 1.01)	0.811	-	-	

Physician global assessments	Publication year	Before 2010	0.72 (0.42 to 1.01)	<0.001	0.0	0.396	0.098
		2010 or after	0.42 (0.24 to 0.61)	<0.001	0.0	0.962	
	Mean age (year)	≥ 55.0	0.54 (0.34 to 0.74)	<0.001	10.1	0.349	0.638
		< 55.0	0.44 (0.12 to 0.76)	0.008	-	-	
	Percentage male (%)	≥60.0	0.56 (0.20 to 0.92)	0.002	44.5	0.165	0.900
		<60.0	0.50 (0.29 to 0.71)	<0.001	0.0	0.591	
	Time since event (months)	≥24.0	0.51 (0.35-0.67)	<0.001	0.0	0.457	-
		<24.0	-	-	-	-	
	Follow-up duration (weeks)	4	0.86 (0.45 to 1.26)	<0.001	75.4	0.007	0.002
		6	1.16 (0.62 to 1.71)	<0.001	71.0	0.016	
		12	0.49 (0.28 to 0.71)	<0.001	0.0	0.750	
	Study quality	High	0.50 (0.34 to 0.66)	<0.001	2.4	0.393	0.449
		Low	0.87 (-0.08 to 1.82)	0.073	-	-	

Disability assessment scale	Publication year	Before 2010	-0.32 (-0.44 to -0.20)	<0.001	0.0	0.732	0.561
		2010 or after	-0.26 (-0.43 to -0.09)	0.003	0.0	0.335	
	Mean age (year)	≥ 55.0	-0.33 (-0.44 to -0.21)	<0.001	0.0	0.737	0.377
		< 55.0	-0.22 (-0.42 to -0.02)	0.030	0.0	0.480	
	Percentage male (%)	≥60.0	-0.26 (-0.43 to -0.09)	0.003	0.0	0.335	0.561
		<60.0	-0.32 (-0.44 to -0.20)	<0.001	0.0	0.732	
	Time since event (months)	≥24.0	-0.30 (-0.40 to -0.20)	<0.001	0.0	0.641	0.649
		<24.0	-0.46 (-1.15 to 0.23)	0.191	-	-	
	Follow-up duration (weeks)	4	-0.33 (-0.63 to -0.03)	0.030	59.2	0.118	0.119
		6	-0.42 (-0.49 to -0.34)	<0.001	0.0	0.461	
		12	-0.30 (-0.40 to -0.20)	<0.001	0.0	0.641	
	Study quality	High	-0.30 (-0.40 to -0.20)	<0.001	0.0	0.756	-
		Low	-	-	-	-	

**Table 3:** Subgroup analysis for lower limb spasticity.

Outcomes	Factors	Groups	SMD and 95% CI	P value	Heterogeneity (%)	P value for heterogeneity	P value for meta-regression
Muscle tone	Publication year	Before 2010	-0.21 (-1.03 to 0.62)	0.618	-	-	0.762
		2010 or after	-0.10 (-0.94 to 0.73)	0.808	92.3	<0.001	
	Mean age (year)	≥ 55.0	-0.69 (-2.08 to 0.70)	0.332	94.0	<0.001	<0.001
		< 55.0	0.31 (-0.12 to 0.74)	0.156	45.8	0.158	
	Percentage male (%)	≥60.0	0.18 (-0.16 to 0.51)	0.298	0.3	0.317	0.037
		<60.0	-0.22 (-1.43 to 0.98)	0.714	94.1	<0.001	
	Time since event (months)	≥24.0	-0.21 (-1.03 to 0.62)	0.618	-	-	0.762
		<24.0	-0.10 (-0.94 to 0.73)	0.808	92.3	<0.001	
	Follow-up duration (weeks)	4	0.62 (0.01 to 1.22)	0.045	85.1	<0.001	0.049
		8	-0.15 (-0.59 to 0.29)	0.504	-	-	
		12	0.02 (-0.77 to 0.80)	0.968	91.6	<0.001	
		> 12	0.02 (-0.46 to 0.50)	0.934	-	-	
	Study quality	High	0.43 (-0.02 to 0.88)	0.058	47.8	0.166	<0.001
		Low	-0.54 (-1.53 to 0.44)	0.278	88.7	<0.001	

Fugl-Meyer score	Publication year	Before 2010	1.20 (-1.88 to 4.28)	0.445	-	-	<0.001
		2010 or after	6.26 (3.39 to 9.12)	<0.001	94.8	<0.001	
	Mean age (year)	≥ 55.0	6.26 (3.39 to 9.12)	<0.001	94.8	<0.001	<0.001
		< 55.0	1.20 (-1.88 to 4.28)	0.445	-	-	
	Percentage male (%)	≥60.0	0.57 (-1.44 to 2.57)	0.580	0.0	0.595	<0.001
		<60.0	8.46 (7.90 to 9.02)	<0.001	27.8	0.239	
	Time since event (months)	≥24.0	1.20 (-1.88 to 4.28)	0.445	-	-	<0.001
		<24.0	6.26 (3.39 to 9.12)	<0.001	94.8	<0.001	
	Follow-up duration (weeks)	4	0.96 (-0.27 to 2.18)	0.125	70.2	0.018	<0.001
		8	-0.56 (-0.89 to -0.23)	0.001	0.0	0.622	
		12	6.66 (4.82 to 8.50)	<0.001	91.0	<0.001	
		> 12	9.04 (7.89 to 10.19)	<0.001	-	-	
	Study quality	High	-	-	-	-	-
		Low	5.09 (2.16 to 8.01)	0.001	94.9	<0.001	

Gait speed	Publication year	Before 2010	-0.00 (-0.04 to 0.03)	0.806	0.0	0.645	0.003
		2010 or after	0.13 (-0.02 to 0.28)	0.089	85.2	0.001	
	Mean age (year)	≥ 55.0	0.13 (-0.03 to 0.29)	0.116	89.9	<0.001	0.129
		< 55.0	-0.01 (-0.06 to 0.04)	0.712	0.0	0.712	
	Percentage male (%)	≥60.0	0.06 (-0.07 to 0.20)	0.343	85.8	0.001	0.016
		<60.0	0.17 (0.06 to 0.28)	0.002	-	-	
	Time since event (months)	≥24.0	-0.02 (-0.09 to 0.06)	0.601	-	-	0.211
		<24.0	0.09 (-0.01 to 0.19)	0.087	85.5	<0.001	
	Follow-up duration (weeks)	4	0.01 (-0.02 to 0.04)	0.533	0.0	0.442	0.454
		8	0.07 (-0.03 to 0.18)	0.158	79.7	0.002	
		12	0.02 (-0.04 to 0.09)	0.434	66.9	0.029	
	Study quality	High	0.00 (-0.04 to 0.04)	1.000	0.0	1.000	0.014
		Low	0.12 (-0.04 to 0.29)	0.139	87.7	<0.001	

**Table 4:** Publication bias assessment for investigated outcomes.

Limb spasticity	Outcomes	P value for Egger	P value for Begg
Upper	Muscle tone	0.591	0.409
	Active upper limb function	0.467	1.000
	Physician global assessments	0.136	0.133
	Disability assessment scale	0.370	0.462
	Adverse events	0.081	0.064

Lower	Muscle tone	0.838	0.806
	Fugl-Meyer score	0.226	0.308
	Gait speed	0.136	0.043
	Adverse events	0.209	0.734

## Data Availability

The data set supporting the results of this article is included within the article.

## Supplementary Materials

Supplementary 1 Search strategies in PubMed.

Supplementary 2 Data set supporting the results of this article.
