# Supplementary material for: Efficacy and Safety of Botulinum Toxin Type A for Limb Spasticity after Stroke: A Meta-Analysis of Randomized Controlled Trials
Source: Biomed Res Int. 2019 Apr 7;2019:8329306. doi: 10.1155/2019/8329306 (PMC6475544; doi:10.1155/2019/8329306)
Supplement: Supplementary 1 — Search strategies in PubMed. [file 8329306.f1.pdf]

Search strategy in PubMed:

#1 "Botulinum Toxin, Type A"[Mesh]

#2 "Abobotulinum toxin A"

#3 "BoNT serotype A"

#4 "Botox a"

#5 "botulin A"

#6 "botulin toxin a"

#7 "botulinum a"

#8 "botulinum neurotoxin a"

#9 "botulinum toxin type A"

#10 "botulinum toxins, type A"

#11 BTXA

#12 "botulinum neurotoxin type A"

#13 "botulinum type A neurotoxin"

#14 "incobotulinum toxin A"

#15 "incobotulinum toxin A"

#16 or/#1-#15

#17 spasticity [Mesh]

#18 spastic

#19 spasmodic

#20 or/#17-#19

#21 #16 and #20
