# Supplementary material for: Efficacy and Safety of Botulinum Toxin Type A for Limb Spasticity after Stroke: A Meta-Analysis of Randomized Controlled Trials
Source: Biomed Res Int. 2019 Apr 7;2019:8329306. doi: 10.1155/2019/8329306 (PMC6475544; doi:10.1155/2019/8329306)
Supplement: Supplementary 2 — Data set supporting the results of this article. [file 8329306.f2.pdf]

upper limb

study	smd	smdl	smdu	group
Simpson 1996	-1.26	-2.26	-0.27	4 week
Simpson 1996	-0.99	-1.95	-0.03	6 week
Simpson 1996	-0.51	-1.43	0.4	8 week
Simpson 1996	0.07	-0.83	0.97	>12 week
Hesse 1998	-0.67	-1.83	0.5	4 week
Hesse 1998	-0.58	-1.74	0.58	6 week
Hesse 1998	-0.5	-1.65	0.65	12 week
Brashear 2002	-0.93	-1.3	-0.56	6 week
Brashear 2002	-0.66	-1.02	-0.3	12 week
Childers 2004	-1.2	-1.83	-0.58	4 week
Childers 2004	-1.13	-1.75	-0.51	6 week
Childers 2004	-1.01	-1.62	-0.39	8 week
Simpson 2009	-1.05	-1.72	-0.38	4 week
Meythaler 2009	0	-0.86	0.86	6 week
Meythaler 2009	-0.27	-1.14	0.59	12 week
Mccrory 2009	-0.99	-1.43	-0.56	8 week
Mccrory 2009	-1.11	-1.55	-0.66	>12 week
Kaji 2010	-0.63	-1.11	-0.14	4 week
Kaji 2010	-0.73	-1.24	-0.23	8 week
Kaji 2010	-0.68	-1.18	-0.18	12 week
Rosales 2012	-1.31	-1.65	-0.97	4 week
Gracies 2015	-1.24	-1.58	-0.9	4 week
Gracies 2015	-0.74	-1.07	-0.41	12 week
Elovic 2016	-0.52	-0.78	-0.26	4 week
Prazeres 2018	0.11	-0.74	0.97	12 week
Prazeres 2018	-0.36	-1.22	0.51	>12 week

Muscle tone

study	smd	smdl	smdu	publication year	mean age	percentage male	time since event	study quality
Simpson 1996	0.07	-0.83	0.97	<2010	>55	<60	>24	low
Hesse 1998	-0.5	-1.65	0.65	<2010	<55	>60	<24	low
Brashear 2002	-0.66	-1.02	-0.3	<2010	>55	<60	>24	high
Childers 2004	-1.01	-1.62	-0.39	<2010	>55	>60	>24	high
Simpson 2009	-1.05	-1.72	-0.38	<2010	<55	<60	<24	high
Meythaler 2009	-0.27	-1.14	0.59	<2010	<55	>60	<24	high
Mccrory 2009	-1.11	-1.55	-0.66	<2010	>55	>60	>24	high
Kaji 2010	-0.68	-1.18	-0.18	>2010	>55	>60	>24	high
Rosales 2012	-1.31	-1.65	-0.97	>2010	>55	>60	<24	high
Gracies 2015	-0.74	-1.07	-0.41	>2010	<55	>60	>24	high
Elovic 2016	-0.52	-0.78	-0.26	>2010	>55	<60	>24	high
Prazeres 2018	-0.36	-1.22	0.51	>2010	<55	>60	>24	high

study	smd	smdl	smdu	group
Simpson 1996	0.95	-0	1.91	4 week
Simpson 1996	2.49	1.26	3.72	6 week
Simpson 1996	0.11	-0.79	1.01	12 week
Brashear 2002	0.54	0.19	0.9	4 week
Brashear 2002	0.94	0.57	1.31	6 week
Kaji 2010	0.48	-0	0.96	4 week
Kaji 2010	0.66	0.15	1.16	6 week
Kaji 2010	0.22	-0.27	0.71	12 week

active upper limb function

study	smd	smdl	smdu	publication year	mean age	percentage male	time since event	study quality
Simpson 1996	0.11	-0.79	1.01	<2010	>55	<60	>24	low
Brashear 2002	0.94	0.57	1.31	<2010	>55	<60	>24	high
Kaji 2010	0.22	-0.27	0.71	>2010	>55	>60	>24	high

study	smd	smdl	smdu	group
Simpson 1996	2.14	0.99	3.29	6 week
Simpson 1996	0.87	-0.08	1.82	12 week
Brashear 2002	1.39	1	1.78	6 week
Brashear 2002	0.58	0.22	0.94	12 week
Childers 2004	1.01	0.39	1.62	4 week
Childers 2004	1.06	0.44	1.67	6 week
Kaji 2010	0.95	0.45	1.44	4 week
Kaji 2010	0.53	0.03	1.02	6 week
Kaji 2010	0.36	-0.13	0.85	12 week
Gracies 2015	1.14	0.8	1.48	4 week
Gracies 2015	0.44	0.12	0.77	12 week
Elovic 2016	0.43	0.17	0.69	4 week

physician global assessments

study	smd	smdl	smdu	publication year	mean age	percentage male	time since event	study quality
Simpson 1996	0.87	-0.08	1.82	<2010	>55	<60	>24	low
Brashear 2002	0.58	0.22	0.94	<2010	>55	<60	>24	high
Childers 2004	1.06	0.44	1.67	<2010	>55	>60	>24	high
Kaji 2010	0.36	-0.13	0.85	>2010	>55	>60	>24	high
Gracies 2015	0.44	0.12	0.77	>2010	<55	>60	>24	high
Elovic 2016	0.43	0.17	0.69	>2010	>55	<60	>24	high

study	rr	rrl	rru	ADR
Smith 2000	1.75	0.1	32.18	
Bakheit 2000	0.6	0.17	2.19	
Bhakta 2000	0.67	0.12	2.57	
Childers 2004	4.5	0.26	78.59	
Yelnik 2007	3	0.37	24.17	
Mccrory 2009	0.58	0.14	2.47	
Kanovsky 2009	0.68	0.12	3.98	
Kaji 2010	0.69	0.16	2.9	
Rosales 2012	2.07	0.39	11.02	
Wolf 2012	3.23	0.14	72.46	
Gracies 2015	3.25	0.75	14.06	
Elovic 2016	2.04	0.44	9.43	

study	smd	smdl	smdu	group
Bhakta 2000	-0.4	-0.48	-0.32	6 week
Bhakta 2000	-0.3	-0.43	-0.17	12 week
Brashear 2002	-0.63	-0.9	-0.36	6 week
Brashear 2002	-0.42	-0.74	-0.1	12 week
Simpson 2009	-0.46	-1.15	0.23	6 week
Kaji 2010	-0.51	-0.83	-0.19	4 week
Kaji 2010	-0.41	-0.75	-0.07	6 week
Kaji 2010	-0.38	-0.68	-0.08	12 week
Gracies 2015	-0.2	-0.42	0.02	4 week
Gracies 2015	-0.2	-0.41	0.01	12 week

disability assessment scale

study	smd	smdl	smdu	publication year	mean age	percentage male	time since event	study quality
Bhakta 2000	-0.3	-0.43	-0.17	<2010	>55	<60	>24	high
Brashear 2002	-0.42	-0.74	-0.1	<2010	>55	<60	>24	high
Simpson 2009	-0.46	-1.15	0.23	<2010	<55	<60	<24	high
Kaji 2010	-0.38	-0.68	-0.08	>2010	>55	>60	>24	high
Gracies 2015	-0.2	-0.41	0.01	>2010	<55	>60	>24	high

lower limb

study	smd	smdl	smdu	group
Burbaud 1996	2.66	1.48	3.84	4 week
Burbaud 1996	-0.21	-1.03	0.62	12 week
Kaji 2010	0.63	0.27	1	4 week
Kaji 2010	0.25	-0.11	0.61	12 week
Fietzek 2014	0.78	0.22	1.35	4 week
Fietzek 2014	0.72	0.16	1.28	12 week
Ding 2015	0.12	-0.36	0.59	4 week
Ding 2015	0.7	0.21	1.19	12 week
Ding 2015	0.02	-0.46	0.49	>12
Ding 2017	-0.24	-0.68	0.2	4 week
Ding 2017	-0.15	-0.59	0.29	8 week
Ding 2017	-1.4	-1.89	-0.91	12 week

Muscle tone

study	smd	smdl	smdu	publication year	mean age	percentage male	time since event	study quality
Burbaud 1996	-0.21	-1.03	0.62	<2010	<55	>60	>24	low
Kaji 2010	0.25	-0.11	0.61	>2010	<55	>60	<24	high
Fietzek 2014	0.72	0.16	1.28	>2010	<55	<60	<24	high
Ding 2015	0.02	-0.46	0.49	>2010	>55	<60	<24	low
Ding 2017	-1.4	-1.89	-0.91	>2010	>55	<60	<24	low

study	smd	smdl	smdu	group
Burbaud 1996	1.5	-1.61	4.61	4 week
Burbaud 1996	1.2	-1.88	4.28	12 week
Tao 2015	2.4	1.16	3.64	4 week
Tao 2015	0.1	-2.54	2.74	8 week
Ding 2015	-0.4	-2.28	1.48	4 week
Ding 2015	7.59	6.73	8.45	12 week
Ding 2015	9.04	7.89	10.19	>12
Ding 2017	0.47	0.13	0.81	4 week
Ding 2017	-0.57	-0.9	-0.24	8 week
Ding 2017	8.32	7.98	8.66	12 week

Fugl-Meyer score

study	smd	smdl	smdu	publication year	mean age	percentage male	time since event	study quality
Burbaud 1996	1.2	-1.88	4.28	<2010	<55	>60	>24	low
Tao 2015	0.1	-2.54	2.74	>2010	>55	>60	<24	low
Ding 2015	9.04	7.89	10.19	>2010	>55	<60	<24	low
Ding 2017	8.32	7.98	8.66	>2010	>55	<60	<24	low

study	smd	smdl	smdu	group
Burbaud 1996	0.06	-0.01	0.13	4 week
Burbaud 1996	-0.02	-0.09	0.06	12 week
Pittock 2003	0	-0.04	0.05	4 week
Pittock 2003	0.01	-0.03	0.05	8 week
Pittock 2003	0	-0.04	0.04	12 week
Kaji 2010	-0.01	-0.07	0.04	4 week
Kaji 2010	-0.03	-0.16	0.11	8 week
Kaji 2010	0	-0.08	0.07	12 week
Tao 2015	0.24	0.12	0.36	8 week
Ding 2017	0.02	-0.09	0.13	4 week
Ding 2017	0.09	-0.01	0.19	8 week
Ding 2017	0.17	0.06	0.28	12 week

Gait speed

study	smd	smdl	smdu	publication year	mean age	percentage male	time since event	study quality
Burbaud 1996	-0.02	-0.09	0.06	<2010	<55	>60	>24	low
Pittock 2003	0	-0.04	0.04	<2010	>55	.	<24	high
Kaji 2010	0	-0.08	0.07	>2010	<55	>60	<24	high
Tao 2015	0.24	0.12	0.36	>2010	>55	>60	<24	low
Ding 2017	0.17	0.06	0.28	>2010	>55	<60	<24	low

study	event	nevent	cevent	cnevent
Pittock 2003	52	127	16	39
Kaji 2010	7	51	7	55
Dunne 2012	11	43	11	18
Wein	154	77	118	108
Kaji 2010	7	51	7	55
Dunne 2012	11	43	11	18
Wein	154	77	118	108

ADR
